# Structural and Functional Characterization of Cargo-Binding Sites on the μ4-Subunit of Adaptor Protein Complex 4

**DOI:** 10.1371/journal.pone.0088147

**Published:** 2014-02-03

**Authors:** Breyan H. Ross, Yimo Lin, Esteban A. Corales, Patricia V. Burgos, Gonzalo A. Mardones

**Affiliations:** Instituto de Fisiología, Facultad de Medicina, and Centro de Investigación Sur-Austral en Enfermedades del Sistema Nervioso, Universidad Austral de Chile, Valdivia, Chile; University of Geveva, Switzerland

## Abstract

Adaptor protein (AP) complexes facilitate protein trafficking by playing key roles in the selection of cargo molecules to be sorted in post-Golgi compartments. Four AP complexes (AP-1 to AP-4) contain a medium-sized subunit (μ1-μ4) that recognizes YXXØ-sequences (Ø is a bulky hydrophobic residue), which are sorting signals in transmembrane proteins. A conserved, canonical region in μ subunits mediates recognition of YXXØ-signals by means of a critical aspartic acid. Recently we found that a non-canonical YXXØ-signal on the cytosolic tail of the Alzheimer's disease amyloid precursor protein (APP) binds to a distinct region of the μ4 subunit of the AP-4 complex. In this study we aimed to determine the functionality of both binding sites of μ4 on the recognition of the non-canonical YXXØ-signal of APP. We found that substitutions in either binding site abrogated the interaction with the APP-tail in yeast-two hybrid experiments. Further characterization by isothermal titration calorimetry showed instead loss of binding to the APP signal with only the substitution R283D at the non-canonical site, in contrast to a decrease in binding affinity with the substitution D190A at the canonical site. We solved the crystal structure of the C-terminal domain of the D190A mutant bound to this non-canonical YXXØ-signal. This structure showed no significant difference compared to that of wild-type μ4. Both differential scanning fluorimetry and limited proteolysis analyses demonstrated that the D190A substitution rendered μ4 less stable, suggesting an explanation for its lower binding affinity to the APP signal. Finally, in contrast to overexpression of the D190A mutant, and acting in a dominant-negative manner, overexpression of μ4 with either a F255A or a R283D substitution at the non-canonical site halted APP transport at the Golgi apparatus. Together, our analyses support that the functional recognition of the non-canonical YXXØ-signal of APP is limited to the non-canonical site of μ4.

## Introduction

Adaptor protein complex 4 (AP-4) is part of a five-member family of heterotetrameric adaptor protein (AP) complexes, AP-1 to AP-5, known for their ability to recognize sorting signals in the cytosolic domain of transmembrane proteins destined to post-Golgi compartments [1]–[4]. AP complexes AP-1 (γ, β1, μ1, σ1), AP-2 (α, β2, μ2, σ2), and AP-3 (δ, β3, μ3, σ3) (subunit composition in parenthesis) are components of protein coats that, after signal recognition, incorporate cargo proteins from a donor compartment into clathrin coated vesicles for transfer to a different compartment [5]. Less well known are AP-4 (ε, β4, μ4, σ4) and the recently discovered AP-5 (ζ, β5, μ5, σ5), but it is expected that they have similar functions in vesicular transport as their counterparts [6]. The five AP complexes are broadly expressed among eukaryotes, with orthologues found in the genome of all metazoan analyzed thus far, as well as of the plant *Arabidopsis thaliana* [6], [7]. The yeast *Saccharomyces cerevisiae*, on the other hand, expresses only AP-1, AP-2 and AP-3 [6], [8], suggesting that AP-4 and AP-5 possess specific roles in higher eukaryotes. Some AP complexes occur as cell-specific isoforms, such as mammalian AP-1B and AP-3B found in epithelia and neurons, respectively [8]. In mice, gene ablation of either of the ubiquitously expressed γ and µ1A subunit of AP-1A or the μ2 subunit of AP-2 is embryonically lethal [9]. In humans, mutations in several subunits of the AP complexes result in severe genetic disorders, such as MEDNIK syndrome for AP-1 [10], Hermansky-Pudlack syndrome for AP-3 [11], cerebral palsy for AP-4 [12], [13], and hereditary spastic paraplegia for AP-5 [4], highlighting the fundamental role that AP complexes play. Each AP complex associates to a characteristic cellular membrane to perform its function. AP-2 is well regarded as cell surface-bound, where it cooperates during clathrin-mediated endocytosis [14]. All of the other AP complexes are found at intracellular membranes. In the case of AP-4 it localizes to the *trans*-Golgi network (TGN; [1], [2], [15]), from where it seems to participate in several sorting events, such as to the basolateral membrane in polarized epithelial cells [16], to the somatodendritic domain in neurons [17], and to early endosomes [18].

The best-characterized sorting signals recognized by AP complexes are comprised of arrays of amino acids that fit one of two consensus motifs: a [DE]XXXL[LIM] signal and a tyrosine-based, YXXØ signal (where Ø is an amino acid with a bulky hydrophobic side chain) [14], [19]. AP-1, AP-2 and AP-3 contain a binding site for [DE]XXXL[LIM] signals that is made up of two subunits, γ and σ1 for AP-1, α and σ2 for AP-2, and δ and σ3 for AP-3 [20]–[22], whereas YXXØ signals bind the C-terminal domain of the μ1, μ2 and μ3 subunit of the respective AP complex [23], [24]. The recognition of these signals is important in the sorting of many type-I, type-II, and multispanning transmembrane proteins [19], and for a variety of cell processes, such as the downregulation of the human immunodeficiency virus coreceptor CD4 that contains a [DE]XXXL[LIM] signal [25], or the efficient endocytosis of the transferrin receptor that contains a YXXØ signal [26]. The structural bases for these interactions have been elucidated by X-ray crystallography of [DE]XXXL[LIM] signals bound to AP-2 [27], and of YXXØ signals bound to either μ2 [28], [29] or μ3A [30]. The crystallographic analyses of the μ subunits show that the C-terminal domain, containing the binding site for YXXØ signals, consists of an immunoglobulin-like fold with sixteen β-strands organized in two β-sandwich subdomains called A and B. The binding site in both μ2 and μ3A is located in subdomain A, where two hydrophobic pockets are shaped on strands β1 and β16 to accommodate the Y and Ø residues of the YXXØ signals [28], [30].

In contrast to AP-1, AP-2, and AP-3, the analysis of AP-4 has shown that it is not capable of effectively binding canonical [DE]XXXL[LIM] and YXXØ signals [31], [32]. The only reported canonical interactions are of the μ4 subunit of AP-4 with YXXØ signals from the lysosomal transmembrane proteins CD63, LAMP-1, and LAMP-2a [2], [31], [33]. These interactions, however, are very weak, and disruption of μ4 expression does not affect the localization of these proteins to lysosomes [16], [32]. A stronger and more functional interaction is one recently found between μ4 and a YXXØ-type, YX[FYL][FL]E motif contained in the cytosolic tail of the Alzheimer's disease amyloid precursor protein (APP) [18]. The YX[FYL][FL]E motif highly resembles canonical YXXØ signals, but it has unique features, and, importantly, disruption of the μ4-YX[FYL][FL]E signal interaction produces a mislocalization of APP from early endosomes to the TGN [18]. This interaction has been characterized by X-ray crystallography [18], and it differs from that of μ2 and μ3A with canonical YXXØ signals. Unexpectedly, the binding site for the YX[FYL][FL]E signal of APP is located on subdomain A of the C-terminal domain of μ4, but at the opposite face of the predicted YXXØ binding site. The Tyr, [FYL], and [FL] residues are fitted on distinct hydrophobic pockets formed by strands β4, β5 and β6, and amino acid substitutions in this site abolish the binding of the APP tail to μ4 [18]. That mutations in the predicted canonical binding site of μ4 abolish the interaction to the YXXØ signal of LAMP-2a [31], suggested the intriguing possibility that μ4 has two binding sites for structurally related tyrosine-based sorting signals. Thus, we decided to analyze in more detail the functionality of both binding sites in the recognition of the YX[FYL][FL]E signal of APP. Here we report that although mutations in either of the tyrosine-based signal binding sites of μ4 affected its interaction to the APP cytosolic tail, only mutations at the YX[FYL][FL]E binding site resulted in the mislocalization of APP to the TGN, indicating no functional role of the canonical site for the recognition of the APP signal.

## Experimental Procedures

### Recombinant DNAs, Site-directed Mutagenesis and Y2H Assays

The cloning of the tail of APP (residues 649–695), full-length human μ4, and all the other yeast two-hybrid (Y2H) constructs, and the cloning of the C-terminal domain of μ4 for expression in *E. coli* was described previously [18]. To generate a construct for mammalian expression, full-length human μ4 was obtained by PCR amplification and cloned into the *Eco*RI and *Sal*I sites of pEGFP-N1 (BD BiosciencesClontech, Mountain View, CA) including a stop-codon before the GFP coding sequence. The cloning of APP-GFP carrying the double mutation F615P/D664A was described previously [34]. Single amino acid substitutions were introduced using the QuikChange mutagenesis kit (Stratagene, La Jolla, CA). Y2H assays were performed as previously described [30]. A set of pCI-neo (Promega) constructs encoding hemagglutinin (HA)-epitope tagged wild-type or mutants of human μ4 was a generous gift of J. Bonifacino and R. Mattera (Cell Biology and Metabolism Program, NICHD, NIH). The nucleotide sequences of all recombinant constructs were confirmed by dideoxy sequencing.

### Expression and Purification of μ4 C-terminal domain variants

Recombinant μ4 C-terminal domain variants tagged with an N-terminal glutathione S-transferase (GST) followed by a TEV protease cleavage site were expressed and purified as previously described [18], with some modifications. Briefly, expression in *E. coli* B834(DE3)pLysS (Novagen, Madison, WI) was induced with 0.2 mM IPTG at 16°C for 36 h. Pellets were resuspended in 50 mM Tris-HCl (pH 8.0), 0.5 M NaCl, 5 mM β-mercaptoethanol, and protease inhibitors (Sigma), and lysed by sonication. The clarified supernatant was purified on glutathione-Sepharose 4B (GE Healthcare). After removal of the GST moiety by TEV cleavage, and sequential passage through glutathione-Sepharose 4B and Ni-NTA (QIAGEN) resins, the C-terminal domain of μ4 was further purified on a Superdex 200 column (GE Healthcare).

### Isothermal Titration Calorimetry

Recombinant μ4 C-terminal variants were dialyzed overnight at 4°C against excess ITC buffer (50 mM Tris-HCl, pH 7.4, 150 mM NaCl), and an APP peptide (ENPTYKFFEQ), a CD63 peptide (SGYEVM), or a TGN38 peptide (SDYQRL; New England Peptide, Gardner, MA) were also prepared in ITC buffer. All ITC experiments were carried out at 28°C using an iTC_200_ instrument (GE Healthcare). Typically, the chamber contained 0.2 ml of 250–500 µM μ4 constructs, and the peptides (2.5–5.0 mM) added in 16 injections of 2.45 µl each. Titration curves were analyzed using Origin software (MicroCal). The binding constant corresponding to each μ4 construct was calculated by fitting the curves to a one-site model.

### Crystallization, Data Collection and Structure Determination

Unless otherwise stated, solutions and crystallization reagents were from Hampton Research (Aliso Viejo, CA). Crystals of the C-terminal domain of μ4-D190A in complex with the APP peptide ENPTYKFFEQ (New England Peptide) were grown by the hanging drop method at 21°C. Prior to crystallization, the protein was incubated at room temperature for 1 h with 2.5 mM peptide. The reservoir solution contained 0.1 M HEPES (pH 7.0) and 15% (w/v) PEG 6000. Hanging drops were set up by mixing 1 µl of reservoir solution with 2 µl of preincubated protein-peptide complex (10 mg/ml). Under these conditions crystals appeared after 24 h. Crystals were cryoprotected in the reservoir solution supplemented with 20% glycerol and then flash-frozen in liquid nitrogen. The complex crystallized in space group *P*2_1_ and crystals diffracted up to 1.84 Å. A native data set was collected from a single crystal at the SER-CAT beamline 22-ID-D, equipped with a MAR CCD detector (Advanced Photon Source, Argonne National Laboratory). Data were processed using HKL2000 [35]. Data collection statistics are shown in Table 1. The structure was determined by molecular replacement with wild-type μ4 without ligands as search model (pdb entry 3L81; [18]) using the program Phaser [36], as implemented in the ccp4 suite of programs for protein crystallography [37]. Iterative manual model building and initial refinement were done using COOT [38] and REFMAC [39]. The final model comprises 269 residues of μ4, the residues TYKFFEQ of APP, and 97 water molecules. Figures were prepared in MacPyMol (The PyMOL Molecular Graphics System, Version 1.2r3pre, Schrödinger, LLC). Crystallographic coordinates and structure factors have been deposited with the Protein Data Bank (accession code 4MDR).

**Table 1:** Statistics of crystallographic data collection and refinement.

**Data Collection**	
Space group	P2_1_
Unit cell parameters	a = 46.5 Å, b = 56.7 Å, c = 60.2 Å, β = 106.7°
Wavelength (Å)	1.0000
Resolution (Å)	1.84 (1.91–1.84)^a^
No. of reflections	115998
No. of unique reflections	25043
I/σ (I)	15.8 (2.7)
Data completeness (%)	97.1 (75.5)
Redundancy	4.6 (2.9)
R_sym_ (%)^b^	7.7 (35.0)
**Structure Refinement**	
R_factor_ (%)	21.35
R_free_ (%)^c^	25.96
r.m.s. bond lengths (Å)	0.022
r.m.s. bond angles	2.046°

a Values in parentheses refer to the highest resolution shell.

b *R*_sym_ = Σ_hkl_ |I_hkl_−<I_hkl_>|/Σ_hkl_ I_hkl_.

c *R*_free_ = free *R*_factor_ based on random 5% of all data.

### Differential Scanning Fluorimetry

The stability of wild-type versus mutant variants of μ4 was assessed by thermal denaturation in the presence of SYPRO orange (Sigma-Aldrich, St. Louis, MO) as previously described [40]. Briefly, 2.8 µg of μ4 or its variants were mixed with 2 µl of a 1∶25 (v/v) dilution of SYPRO orange in a final volume of 20 µl, and the change in fluorescence was monitored over a temperature-range of 25–90°C. Fluorescence was excited and detected at 470 nm and 510 nm, respectively. Samples were run in triplicate on a Rotor-Gene Q real-time rotary analyzer (QIAGEN). The analysis was done with the Rotor-Gene Q software (QIAGEN), and the *T*_m_ values were determined calculating the negative first derivative of the raw data.

### Limited Proteolysis and N-terminal Sequencing

Recombinant μ4 C-terminal variants (7 µg) were subjected to limited proteolysis using 4 µg/ml proteinase K in digestion buffer (25 mM Tris pH 7.4, 150 mM NaCl, 10% glycerol, 5 mM β-mercaptoethanol). Samples were incubated at different temperatures in the range of 25–55°C, and after different periods of time aliquots were taken and the reaction was stopped by addition of 10 mM phenylmethylsulfonyl fluoride (PMSF). The digestion products were analyzed by SDS-PAGE in 4–12% gradient gels using the NuPAGE Bis-Tris gel system (Life Technologies), according to the manufacturer's instructions, and gels were stained with Coomassie Brilliant Blue. Alternatively, protein fragments were separated by SDS-PAGE, blotted to a polyvinylidene fluoride (PVDF) membrane, and analyzed by N-terminal amino acid sequencing applying automated Edman degradation by using a 492 cLC protein sequencer (Applied Biosystems).

### Cell Culture, Transfection and Biochemical Assays

H4 human neuroglioma or MDA-MB-231 human mammary gland epithelial cells obtained from the American Type Culture Collection (Manassas, VA) were maintained in DMEM (H4) or DMEM/F12 (MDA-MB-231) supplemented with 10% (v/v) fetal bovine serum (FBS), 100 U/ml penicillin and 100 µg/ml streptomycin (Life Technologies). Transfections were carried out using Lipofectamine 2000 (Life Technologies) for 1 h at 37°C in the absence of FBS, and cells were analyzed 16, 24 or up to 36-h after transfection, for which we observed no notorious signs of loss in cell viability. SDS-PAGE, immunoblotting, and immunoprecipitation were performed as described [41]. H4 cells after 16 h of transfection were washed twice with cold phosphate buffered saline supplemented with 0.1 mM CaCl_2_ and 1 mM MgCl_2_ (PBS-Ca/Mg), and incubated at 4°C for 1 h in lysis buffer (50 mM Tris-HCl pH 7.4, 150 mM NaCl, 1 mM EDTA, 1% (v/v) Triton X-100, and a cocktail of protease inhibitors). Soluble extracts were subjected to overnight immunoprecipitation at 4°C either with rabbit anti-HA-epitope antibody (kindly provided by R. Hegde, MRC Laboratory of Molecular Biology, Cambridge, UK) or with mouse antibody to the ε subunit of AP-4 (BD Biosciences) immobilized onto protein A- or a 1∶1 mix of protein A- and protein-G Sepharose beads (GE Healthcare), respectively. Soluble extracts and immunoprecipitates were processed by SDS-PAGE and blotted with horseradish peroxidase-conjugated mouse anti-HA antibody (Macs Miltenyi Biotec) or with mouse antibody to the ε subunit of AP-4.

### Immunofluorescence Microscopy and Quantification of Colocalization

Indirect immunofluorescence staining of fixed, permeabilized cells was performed as previously described [42], using sheep polyclonal antibody anti-TGN46 (Serotec), and mouse monoclonal antibody anti-EEA1 (BD Biosciences) or rabbit polyclonal antibody anti-EEA1 (Santa Cruz Biotechnology), followed by the secondary antibodies Alexa-647–conjugated donkey anti-mouse IgG, Alexa-594–conjugated donkey anti-rabbit IgG, or Alexa-594– or -647–conjugated donkey anti-sheep IgG (Life Technologies). Images were acquired either with an Olympus FluoView FV1000 scanning unit fitted on an inverted Olympus IX81 microscope and equipped with a PlanApo 60x oil immersion objective (NA 1.40; Olympus, Melville, NY), using similar settings as described previously [41], or with an AxioObserver.D1 microscope equipped with a PlanApo 63x oil immersion objective (NA 1.4), and an AxioCam MRm digital camera (Carl Zeiss). Quantitative analysis of colocalization was performed as we previously described [18], with minor modifications. Briefly, 12-bit images were acquired under identical settings avoiding signal saturation, and corrected for noise, cross-talk, and background signals on each set of images. The signals of TGN46 or EEA1 in each set of images were used in Image J (version 1.44o; Wayne Rasband, NIH, http://imagej.nih.gov) to define masks regarded as Golgi/TGN or early endosomes localization, respectively. The percentage of localization in each compartment was calculated for each cell (n = 10–15) subtracting either the Golgi/TGN or the early endosomes mask from the total integrated pixel intensity of APP-GFP, and the remainder of the signal was considered as in the ‘rest of the cell’.

### Statistical Analysis

All experiments and measurements were performed at least three times. Data analysis was performed using Microsoft Excel for Mac 2011 (Microsoft Corporation). When appropriate, results were expressed as the mean ± standard deviation. Statistical significance was determined by one-tailed *t*-test. *P*-values of *p*<0.05 (*), *p*<0.01 (**), *p*<0.001 (***) were regarded as statistically significant, and are indicated in the figures.

## Results and Discussion

**Figure 1:**
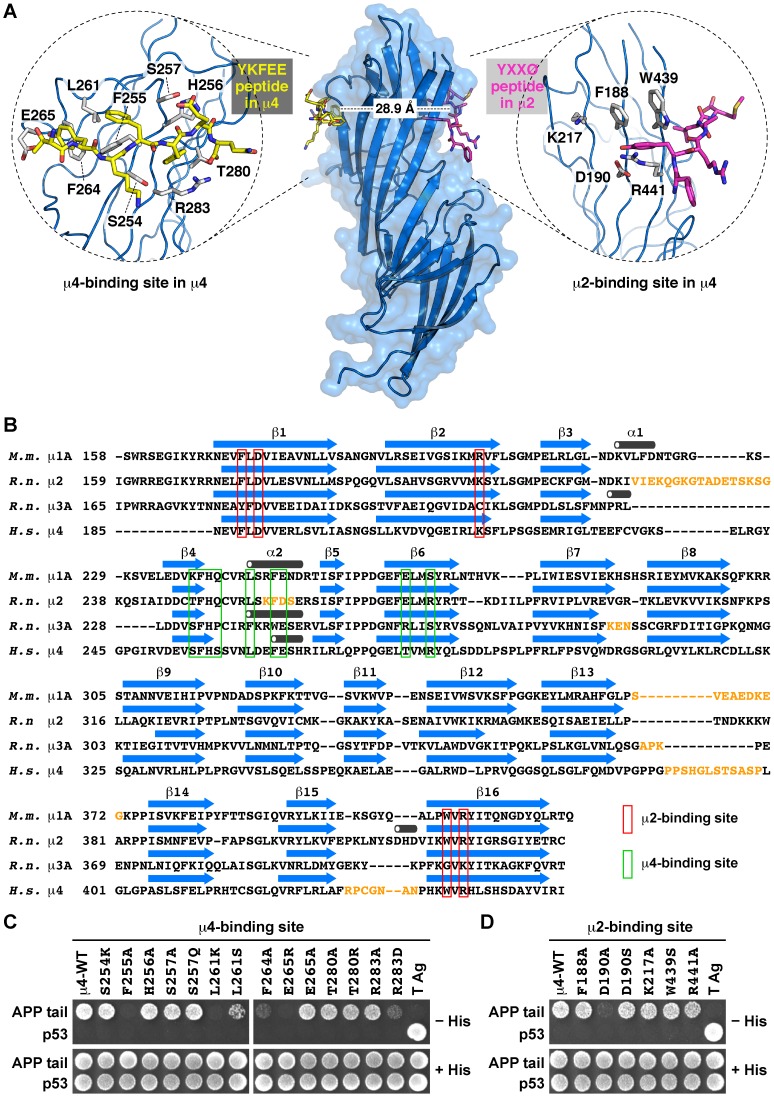
Yeast two-hybrid analysis of the interaction of μ4 with the cytosolic tail of APP. (**A**) Superposition of the surface and ribbon representations of human wild-type μ4 C-terminal domain (pdb entry 3L81). The insets show an enlargement of the μ4-binding site and the putative μ2-binding site with residues chosen for the yeast two-hybrid (Y2H) analysis. The APP peptide (TYKFFEQ; stick model) bound to the μ4-binding site is in yellow, and the EGFR peptide (FYRALM; stick model; pdb entry 1BW8) superposed to the putative μ2-binding site is in magenta. (**B**) Sequence alignment of the C-terminal domain of the μ subunits of known crystal structure depicting critical residues at the corresponding μ2- and μ4-binding sites. Disordered loops are in yellow letters, and arrows and cylinders represent β-strands and α-helices, respectively. *M.m*., *Mus musculus*; *R.n*., *Rattus norvegicus*; *H.s*., *Homo sapiens*. (**C**) and (**D**) Yeast were co-transformed with plasmids encoding Gal4bd fused to the cytosolic tail of the amyloid precursor protein (APP) indicated on the left, and Gal4ad fused to wild-type or mutant μ4 constructs indicated on top of each panel. (**C**) Y2H analysis of μ4 with mutations on the YKFFE binding site (μ4-binding site). (**D**) Y2H analysis of μ4 with mutations on a putative YXXØ binding site (μ2-binding site). Mouse p53 fused to Gal4bd and SV40 large T antigen (T Ag) fused to Gal4ad were used as controls. Co-transformed cells were spotted onto His-deficient (-His) or His-containing (+His) plates and incubated at 30°C. Growth is indicative of interactions. Some mutations on either of the two sites affect the interaction of μ4 with the cytosolic tail of APP.

**Figure 2:**
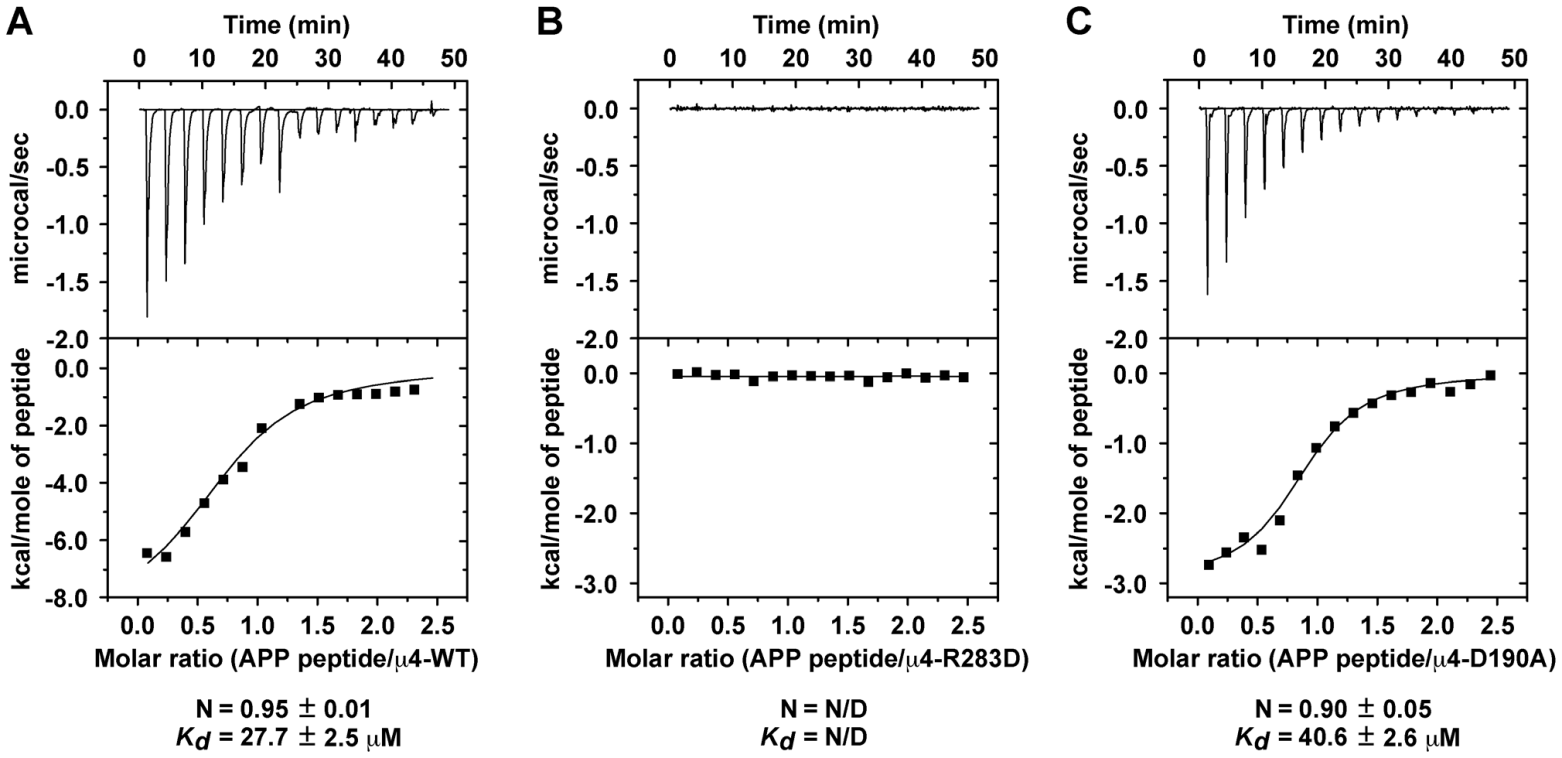
Isotermal titration calorimetry analysis of the interaction of μ4 with the APP sorting signal. Isothermal titration calorimetry of the APP ENPTYKFFEQ peptide with recombinant C-terminal domain of wild-type μ4 (**A**), μ4-D190A (**B**), or μ4-R283D (**C**). The stoichiometry (N) and *K_d_* for the interaction of the ENPTYKFFEQ peptide with either μ4-WT or μ4-D190A are expressed as the mean ± SEM (n = 3). Because the interaction of the ENPTYKFFEQ peptide with μ4-R283D is undetectable the stoichiometry and *K_d_* were not determined (N/D).

Previously, it was found that the YX[FYL][FL]E-type signal from APP (YKFFE) binds to a distinct site on the C-terminal domain of the μ4 subunit of AP-4 [18]. This tyrosine-based signal is related to the YXXØ motif, which is the signal contained in the cytosolic tail of transmembrane proteins that binds to a different site on the μ2 subunit of AP-2 [28], or on the μ3A subunit of AP-3 [30]. In this study, we wanted to determine whether this putative second binding site on μ4, referred here as the μ2-binding site (Figure 1, A and B), played any role in the binding to the YKFFE signal of APP. First, we extended our previous characterization by yeast two-hybrid (Y2H) analysis on the functional role of the binding site of μ4 for APP (referred to as μ4-binding site in the following; Figure 1, A and B). We systematically mutated residues within both binding sites to reveal their individual contribution to the recognition of the YKFFE signal. In addition to a previously identified sensitivity to the mutations F255A, L261S, and R283D [18], we found that the mutations L261K or E265R completely abolished, and F264A drastically reduced, the binding to the YKFEE signal (Figure 1C). However, the mutations S254K, S257Q, T280A, or T280R had little or no effects on signal recognition (Figure 1C), suggesting that although located within the binding pocket, these residues do not contribute significantly to this interaction, similar to the null effect of the mutations H256A, S257A, E265A, and R283A reported previously [18]. To assess the functional role of the μ2-binding site of μ4, we chose to mutate residues that are involved in YXXØ recognition and structurally conserved between the μ2-binding site in μ4 and μ2 or μ3A [30], [31], [43], [44]. The single mutations F188A, D190S, K217A, W439S or R441A, resulted in little or no effect on the interaction of the APP tail with μ4 (Figure 1D). Surprisingly, the single mutation D190A abrogated the interaction of μ4 with the YKFFE signal (Figure 1D). The carboxylate of D190 in μ4 is predicted to establish a critical hydrogen bond with the phenolic hydroxyl group of the Tyr residue of YXXØ signals, interactions seen with D176 in μ2 or D182 in μ3A [28], [30]. A similar role is seen for D174 in μ1A that binds to the Tyr residue of the YXXØ-related signal YSQA in the cytosolic tail of the major histocompatibility complex class I (MHC-I; [45]). Previous reports have shown that μ4 binds weakly to certain canonical YXXØ signals [2], [31], [33], and that the single D190A mutation results in the loss of these interactions [31]. This mutational analysis further validated the identity of the μ4-binding site, but suggested the intriguing possibility that the μ2-binding site also participates in the recognition of the YKFFE signal. Alternatively, because the predicted site in μ4 for YXXØ signals is located on a face opposite to that of the YKFFE signal of APP ([18]; Figure 1A), the D190A mutation might change the conformation of μ4 affecting the μ4-binding site, and/or render μ4 structurally less stable. To discriminate between these two possibilities we first performed additional analysis *in vitro* of the interaction of μ4 with the YKFFE signal by isothermal titration calorimetry using purified components. As we previously showed [18], a synthetic ENPTYKFFEQ peptide derived from the APP tail bound to a single site on recombinant μ4 C-terminal domain with *K_d_* = 27.7±2.5 µM (Figure 2A), and the single mutation R283D at the μ4-binding site rendered the interaction undetectable (Figure 2B). In contrast to what we expected, the single mutation D190A did not preclude the interaction of the μ4 C-terminal domain with the ENPTYKFFEQ peptide, instead the ITC analysis showed a single site on μ4-D190A with *K_d_* = 40.6±2.6 µM (Figure 2C). The mutation resulted in binding with lower affinity (*p*<0.05), sufficient to prevent the interaction as tested by Y2H, suggesting that D190 is not necessary for interaction with the tyrosine residue of the YKFFE signal, but instead that this mutation might alter the conformation and/or the stability of μ4. Consistent with the Y2H analyses, a D190S mutant (μ4-D190S) binds to the ENPTYKFFEQ peptide with similar affinity as the binding of wild-type μ4 (*p*>0.1; Figure S1, A and B). On the other hand, we found no detectable binding of wild-type μ4 to peptides bearing the canonical YXXØ signals of TGN38 or CD63 (Figure S1, C and D), peptides that we previously showed bind well to the C-terminal domain of μ3A [18].

**Figure 3:**
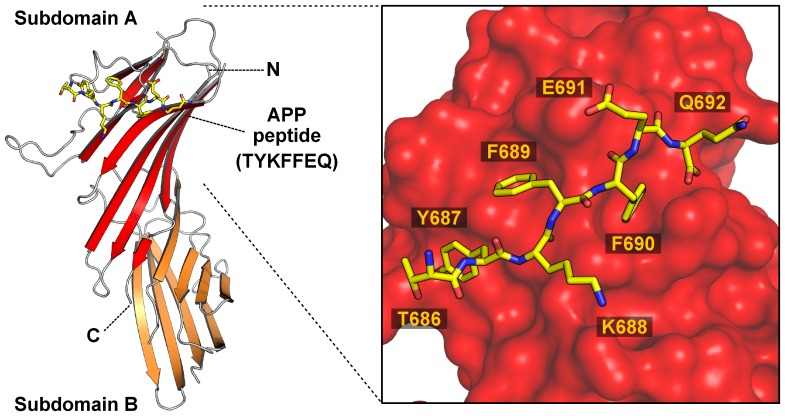
Crystal structure of the μ4-D190A C-terminal domain bound to the APP sorting signal. Ribbon representation of human μ4-D190A C-terminal domain with subdomain A colored red, subdomain B colored orange, and the APP peptide (TYKFFEQ; stick model) colored yellow. The position of the N- and C-termini are indicated. The inset shows the orientation of the APP peptide side chains on the binding site, with atoms of the peptide colored yellow (carbon), red (oxygen), or blue (nitrogen). The crystal structure of μ4-D190A C-terminal domain bound to the APP peptide is very similar to that of wild-type μ4.

**Figure 4:**
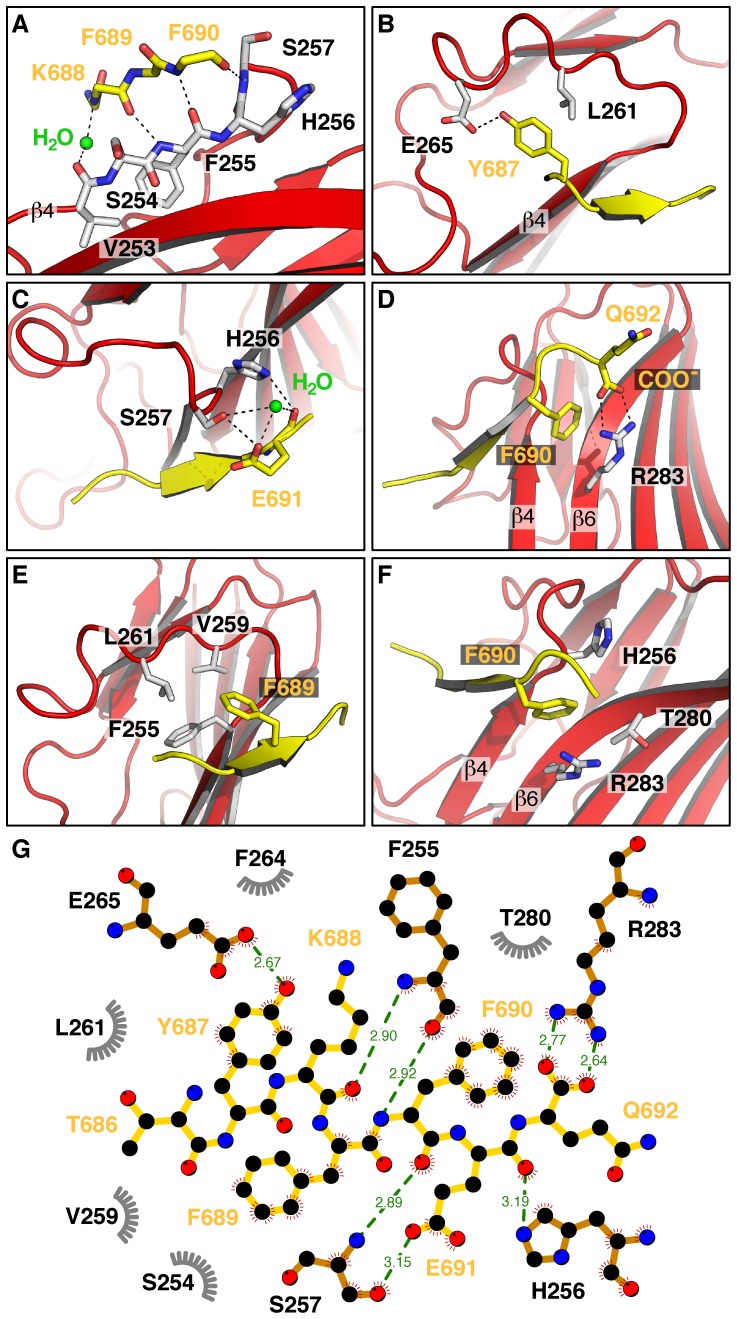
Interaction of the APP peptide with binding site residues on μ4-D190A. (**A**–**G**). Hydrogen-bonds are indicated by dashed lines. (**A**) Direct and water-mediated hydrogen bonding between backbone-residues of β4 (μ4-D190A) and residues 688–690 of the APP peptide. Side-chains of the APP peptide are omitted for clarity. (**B**) The hydroxyl group and the aromatic ring of Tyr-687 in the APP peptide hydrogen-binds Glu-265, and forms a hydrophobic interaction with Leu-261 of μ4-D190A, respectively. (**C**) Glu-691 in the peptide forms hydrogen bonds with His-256 and Ser-257 via its main-chain carbonyl and side chain carboxylate, respectively. (**D**) Phe-689 of APP binds into a hydrophobic groove, formed by the side chains of Phe-255, Val-259, and Leu-261 of μ4-D190A. (**E**) Phe-690 is deeply buried in a cavity formed by the hydrocarbon portions of His-256, Thr-280, and Arg-283 of μ4-D190A. (**F**) The aromatic ring of Phe-690 in the peptide participates in a cation-π interaction with the side-chain of Arg-283 in μ4-D190A. Arg-283 also forms a bidentate salt bridge with the C-terminal carboxylate of the peptide. (**G**) Two-dimensional, schematic representation of the interactions shown in A-F using LigPlot^+^ [51], showing peptide-protein hydrogen bonds in green, and hydrophobic contacts in grey. The numbers of the APP peptide residues are as in APP695.

To determine whether the single D190A mutation changed the conformation of μ4, we solved the crystal structure of the C-terminal domain of μ4-D190A (residues 185–453 of the human protein) in complex with the ENPTYKFFEQ peptide from APP at 1.84 Å resolution (Figure 3; Table 1). Similar to wild type μ4 [18], the μ4-D190A C-terminal domain is organized into two subdomains, A and B, and has an immunoglobulin-like β-sandwich fold comprising 16 strands (Figure 3). The overall crystal structure is virtually identical to that of wild-type μ4, as demonstrated by a root mean square deviation of 0.190 Å for superimposable Cα coordinates. As was seen with wild type μ4, of the ENPTYKFFEQ peptide, only the TYKFFEQ portion was visible in the density map (Figure S2) and, as expected, bound to the μ4-binding site (Figures 3 and S2). The area of the interface between the YKFFE signal and μ4-D190A is 431.1 Å^2^, analogous to that on wild type μ4 that is 430.5 Å^2^, as calculated by the PISA server [46]. The μ4-D190A - YKFFE interface maintained considerable polarity, and all eight direct hydrogen bonds between μ4 and the peptide are preserved (Figure 4; [18]). The residues Y687 to F690 from the peptide are in β conformation, with residues 688–690 forming a β-sheet with residues 253–257 of μ4-D190A (Figure 4A). Of all the stabilizing interactions, Y687 forms with its phenolic hydroxyl one of the shortest hydrogen bonds with the carboxylate of E265 on μ4-D190A (Figure 4, B and G). Both the carbonyl group and the carboxylate of E691 of the APP peptide are forming hydrogen bonds with the side chain of H256, and a water molecule and the side chain of S257, respectively (Figure 4, C and G). Besides hydrogen bonding, the peptide is stabilized by hydrophobic contacts formed by its three aromatic residues: Y687 with the side chain of L261 (Figure 4, B and G); F689 with the side chain of F255, V259, and L261 (Figure 4D); and F690 with the hydrocarbon portions of H256, T280, and R283 (Figure 4E). Finally, the phenyl ring of F690 forms a cation-π interaction with the guanidinium group of R283 on μ4-D190A (Figure 4, F and G). Additionally, Q692, the last residue in the peptide, forms a bidentate salt bridge with its free carboxylate and the guanidinium group of R283 (Figure 4, F and G). Together, this crystallographic analysis demonstrated that μ4-D190A binds the YKFFE signal, and provided evidence against the possibility that even a minor change in conformation was the cause of the weaker interaction.

**Figure 5:**
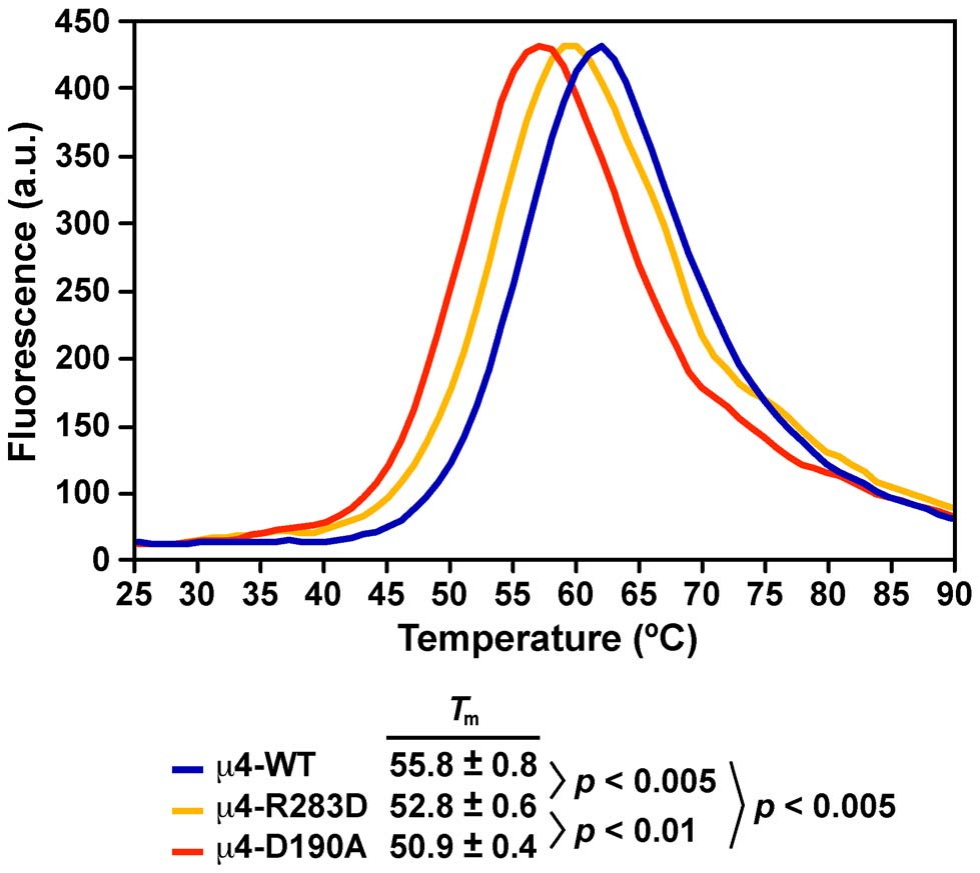
Thermal stability analysis of the C-terminal domain of μ4. The thermal unfolding of the recombinant C-terminal domain of wild-type μ4, μ4-D190A, or μ4-R283D was analyzed by differential scanning fluorimetry following fluorescence changes in the presence of SYPRO Orange. Representative melting curves of each μ4 variant are shown. The calculated *T*_m_ value, defined as the maximum of the first derivative of the raw data, is expressed as the mean ± SD (n = 3).

To investigate further the reason of the lower affinity of μ4-D190A for the YKFFE signal, we determined the thermal stability of recombinant μ4-D190A C-terminal domain using differential scanning fluorimetry (DSF) compared to that of wild-type μ4 and to that of μ4 with the single mutation R283D at the μ4-binding site (μ4-R283D). We measured apparent *T*_m_ values by following the unfolding process during thermal denaturation, and found that the *T*_m_ of μ4-D190A was 50.9±0.4°C, significantly lower than that of wild-type μ4, which was 55.8±0.8°C (Figure 5), indicating that the mutation D190A at the μ2-binding site makes μ4 less stable. The *T*_m_ value of μ4-R283D was 52.8±0.6°C (Figure 5), higher than the *T*_m_ value of μ4-D190A, but lower than that of wild-type μ4, indicating that the mutation R283D at the μ4-binding site produces to some extent less stability in μ4. Yet, we favor a scenario in which the reduced stability of μ4-D190A lowered the affinity for the YKFFE signal. In contrast, and again consistent with the Y2H analysis, μ4-D190S showed similar thermal stability as to wild-type μ4 (Figure S3A).

**Figure 6:**
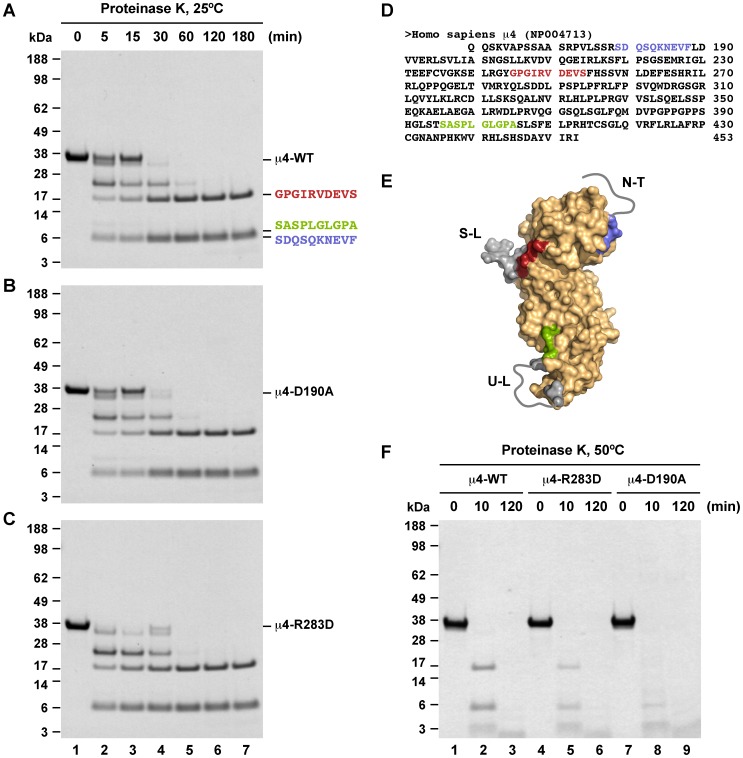
Limited proteolysis analysis of the C-terminal domain of wild-type μ4. Recombinant C-terminal domain of wild-type μ4 (**A**), μ4-D190A (**B**), or μ4-R283D (**C**) were incubated with proteinase K at 25°C at an enzyme:substrate ratio of 1∶100, and after the times indicated on top of the panel the digestion was stopped by addition of PMSF. The reaction products were analyzed by SDS-PAGE and gels stained with Coomassie Brilliant Blue. In this condition similar stable fragments are produced from all μ4 variants. Samples from a similar gel shown in (A) were electroblotted onto a PVDF membrane. The three bands shown in lane 7 were excised and processed for N-terminal sequencing by Edman degradation, and the resulting amino acid sequences are shown on the right. (**D**) Amino acid sequence of the recombinant C-terminal domain of human μ4 (residues 160-453; accession number in parenthesis), with the N-terminal sequence of the fragments shown in (A) highlighted in different colors. (**E**) Surface model of the μ4 C-terminal domain with amino acids of the proteolytic fragments colored as in A and D. The regions digested are colored in grey, corresponding to a structured loop (S–L), an unstructured loop (U–L), and unstructured N-terminal residues (N–T) (represented as grey lines). (**F**) The same μ4 variants were processed as in A to C, but incubated with proteinase K at 50°C at the indicated times on top of the panel. In this case the three μ4 variants have different levels of sensitivity to proteinase K. The position of molecular mass markers is indicated on the left.

To find additional evidence that μ4-D190A was less stable than wild-type μ4, we performed limited proteolysis analysis on the same recombinant variants used in the DSF experiments. Limited proteolysis produces stable intermediates that represent compact regions not further accessible to proteases, and it has been widely used as a method to evaluate protein stability [47]. A time course up to three hours of digestion at 25°C resulted in three major fragments from wild-type μ4 resistant to proteinase K, one with apparent molecular mass of 17 kDa, and a diffuse doublet at 6 kDa (Figure 6A). The same time course of digestion resulted in similar patterns of resistant fragments from μ4-D190A (Figure 6B), and from μ4-R283D (Figure 6C). N-terminal sequencing allowed the unequivocal identification of the first ten amino acids from each of the three fragments as part of the μ4 C-terminal domain (Figure 6D). The fragment with 17 kDa started with the sequence GPGIRVDEVS, and the upper and lower fragments of the doublet at 6 kDa started with the sequence SASPLGLGPA and SDQSQKNEVF, respectively (Figure 6, A–D). A close inspection to the crystal structure of either wild-type μ4 (pdb entry 3l81; [18]) or μ4-D190A revealed that the SDQSQKNEVF sequence is at the end of the disordered N-terminal region, in which only the NEVF portion is visible in the crystal structure (Figure 6E). Likewise, the SASPLGLGPA sequence is part of a disordered loop followed by a well-structured region, with the LGLGPA portion visible in the crystal structure (Figure 6E). Conversely, the GPGIRVDEVS sequence corresponds to a structured region after a loop that is completely visible in the crystal structure (Figure 6E). This analysis is consistent with all three fragments being generated by proteolysis of regions predicted to be accessible. Moreover, the identical cleavage patterns indicate that both the D190A and the R283D mutations did not cause significant conformational changes to μ4. Because with the previous experiment we were unable to distinguish protease sensitivity among the μ4 variants, we performed limited proteolysis with proteinase K for 10 min at 50°C, which is a temperature close to the *T*_m_ values measured by DSF (Figure 5). With this experimental setup we observed for both wild-type μ4 and μ4-R283D, three fragments that likely corresponded to the stable fragments generated at 25°C (Figure 6F). Similarly, the proteolysis pattern of μ4-D190S was indistinguishable from that of wild-type μ4 (Figure S3B). In contrast, μ4-D190A was almost completely degraded after 10 min of incubation with proteinase K (Figure 6F), indicating that the substitution of Ala for Asp-190 at the μ2-binding site makes μ4 less stable than the substitution of Asp for Arg-283 at the μ4-binding site, which is in agreement with the DSF data.

We next examined the functional role of both the μ2- and the μ4-binding site of μ4 on the intracellular trafficking of APP. We have shown that the YKFFE-μ4 interaction is functional *in vivo* because its disruption by depletion of μ4 by RNAi produces a shift in the distribution of APP from endosomes to the TGN [18]. To evaluate now the function of each binding site for tyrosine-based signals, we tested a dominant-negative effect by overexpressing untagged or HA-epitope-tagged full-length wild-type μ4 or the variants untagged or HA-epitope-tagged μ4-D190A, μ4-D190S, μ4-F255A or μ4-R283D in H4 neuroglioma cells, as well as in MDA-MB-231 mammary gland epithelial cells, and examined by immunofluorescence the distribution of APP-GFP. A similar dominant-negative approach has been used to assess the functionality of the μ2-binding site of μ1A and of μ2 in the sorting of the transferrin receptor in rat hippocampal neurons [48], and HeLa cells [44], respectively. APP is a ubiquitously-expressed type-I transmembrane glycoprotein that traffics by the secretory pathway to the cell surface. During transport, it undergoes proteolytic processing by endopeptidases that eventually produce pathogenic Aβ peptide through the so called amyloidogenic pathway [49]. APP is also a substrate of α-secretases in an alternative, more active non-amyloidogenic pathway [49], and of caspase cleavage [50]. To facilitate the analysis, we transfected cells with a plasmid encoding APP-GFP carrying substitutions that we have previously shown to abolish the cleavage by α-secretases and caspases [34].

**Figure 7:**
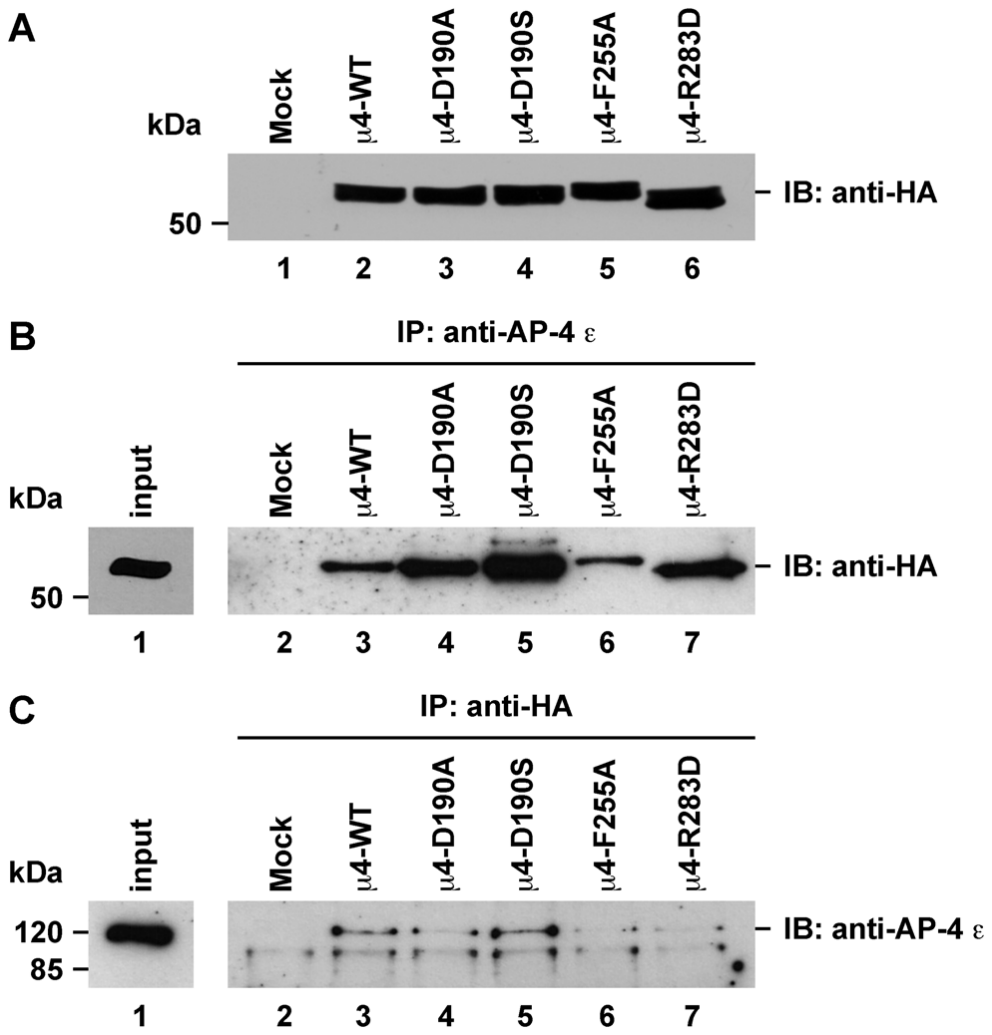
HA-epitope-tagged μ4 variants incorporate into endogenous AP-4 complex. H4 neuroglioma cells were transfected with a plasmid encoding either of the indicated HA-epitope-tagged variants of μ4. After 16-h, cell lysates were prepared and samples were subjected to SDS-PAGE followed by immunoblot with mouse anti-HA-epitope antibody (**A**). Samples of cell lysates were also subjected to immunoprecipitation using mouse antibody to the ε subunit of AP-4 followed by SDS-PAGE and immunoblotting with horseradish peroxidase-conjugated anti-HA-epitope antibody (**B**), or immunoprecipitation using rabbit anti-HA-epitope antibody followed by SDS-PAGE and immunoblotting with mouse antibody to the ε subunit of AP-4 (**C**). The position of molecular mass markers is indicated on the left.

**Figure 8:**
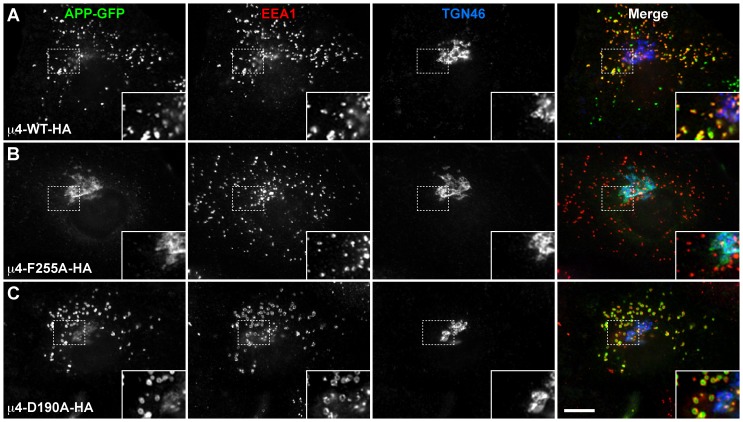
APP redistributes from endosomes to the TGN upon overexpression of μ4-F255A-HA. MD-MB-231 cells were cotransfected with a plasmid encoding either of the indicated HA-epitope-tagged variants of μ4, and with a plasmid encoding APP-GFP carrying the double mutation F615P/D664A. After 24-h cells were fixed, permeabilized, stained for EEA1 and TGN46, and examined by fluorescence microscopy. Merging green, red, and blue channels generated the fourth image on each row; yellow indicates overlapping localization of the green and red channels, cyan indicates overlapping localization of the green and blue channels, magenta indicates overlapping localization of the red and blue channels, and white indicates overlapping localization of the red, green, and blue channels. Insets show 2× magnifications. Bar, 10 µm.

All μ4 constructs expressed at similar levels, either untagged (data not shown) or HA-epitope-tagged (Figure 7A), although we noticed minor differences in the electrophoretic mobility among them likely as a result of the corresponding amino acid substitution. We determined that all HA-epitope-tagged μ4 variants were incorporated into endogenous AP-4 complex, by either immunoprecipitating with antibody to the ε subunit of AP-4 followed by immunoblotting with antibody to the HA epitope (Figure 7B), or immunoprecipitating with antibody to the HA epitope followed by immunoblotting with antibody to the ε subunit of AP-4 (Figure 7C). After 24-h of transfection we observed that unlike overexpression of HA-epitope-tagged wild-type μ4, which produced no apparent change in the distribution of APP that localized mainly in endosomes, as indicated by colocalization with the early endosome marker EEA1 (Figure 8A and Table S1; [18]), overexpression of HA-epitope-tagged μ4-F255A or HA-epitope-tagged μ4-R283D resulted in a strong redistribution of APP from endosomes to the TGN (Figures 8B and S4A, and Table S1), but did not affect the distribution of other transmembrane proteins (data not shown). In contrast, overexpression of HA-epitope-tagged μ4-D190A caused a minor redistribution of APP to the TGN, with the majority of APP localized to endosomes (Figure 8C and Table S1), consistent with the notion that the D190A mutation did not abolish a functional interaction of μ4 with the YKFFE signal of APP. Similar results were observed in cells transfected with untagged μ4 constructs (Figures S4B and S5, and Table S1), indicating that the redistribution of APP-GFP was not an artifact produced by the HA-epitope. Accordingly, overexpression of either HA-epitope-tagged or untagged μ4-D190S produced little or no effect on the localization of APP-GFP in endosomes (Figure S4, C and D, and Table S1). Altogether our mutational, crystallographic, biochemical and functional analyses rule out the μ2-binding site as playing an important role in the recognition of the YKFFE signal, and provides additional evidence for the functionality of the μ4-binding site. Finally, here we establish the use of dominant-negative mutants of μ4 as a useful tool for the study of the function of the AP-4 complex.

## Supporting Information

Figure S1 **Isotermal titration calorimetry analyses of the interaction of μ4-D190S with the APP sorting signal and of μ4 with canonical YXXØ signals.** Isothermal titration calorimetry of the APP ENPTYKFFEQ peptide with recombinant C-terminal domain of wild-type μ4 (**A**), or μ4-D190S (**B**). The stoichiometry (N) and *K_d_* for the interaction of the ENPTYKFFEQ peptide with either μ4-WT or μ4-D190S are expressed as the mean ± SEM (n = 3). Isothermal titration calorimetry of recombinant C-terminal domain of wild-type μ4 with the TGN38 SDYQRL peptide (**C**), or the CD63 SGYEVM peptide (**D**). Because the interaction of wild-type μ4 with either of the peptides with canonical YXXØ signals is undetectable the stoichiometry and *K_d_* were not determined (N/D).
(TIF)

Figure S2 **Comparison of electron density maps of μ4 and μ4-D190A.** (**A**) Difference electron density map of the APP peptide (TYKFFEQ) bound to μ4-D190A C-terminal domain (*F*_o_-*F*_c_ contoured at 3σ, green mesh). The density was calculated after solving the structure by molecular replacement using wild-type μ4 C-terminal domain without ligands as search model. The position of the peptide was revealed by superimposing the search model with the μ4 C-terminal domain bound to the APP peptide (represented in stick model; pdb entry 3L81). (**B**) Negative difference electron density map of μ4-D190A (*F*_o_-*F*_c_ contoured at 3σ, red mesh) at the site of Asp-190 (dotted green circle), observed in the initial electron density as described in (A), superimposed to the electron density map of μ4-D190A (2*F*_o_-*F*_c_ contoured at 2σ, blue mesh) after refining against μ4-D190A. The superimposed structure of 3L81 is shown as sticks. (**C**) Electron density map and stick model of 3L81 at a similar region shown in (A). (**D**) Electron density map and stick model of 3L81 at a similar region shown in (B).
(TIF)

Figure S3 **Thermal stability and limited proteolysis analyses of the C-terminal domain of μ4-D190S.** (**A**) The thermal unfolding of the recombinant C-terminal domain of wild-type μ4, or μ4-D190S was analyzed by differential scanning fluorimetry following fluorescence changes in the presence of SYPRO Orange. Representative melting curves of each μ4 variant are shown. The calculated *T*_m_ value, defined as the maximum of the first derivative of the raw data, is expressed as the mean ± SD (n = 3). (**B**) Samples of recombinant C-terminal domain of wild-type μ4, or μ4-D190S were incubated with proteinase K at 50°C at an enzyme:substrate ratio of 1∶100, and after the times indicated on top of the panel the digestion was stopped by addition of PMSF. The reaction products were analyzed by SDS-PAGE and gels stained with Coomassie Brilliant Blue. The position of molecular mass markers is indicated on the left.
(TIF)

Figure S4 **APP redistributes from endosomes to the TGN upon overexpression of μ4-R283D-HA or μ4-R283D.** MD-MB-231 cells were cotransfected with a plasmid encoding either of the indicated HA-epitope-tagged or untagged variants of μ4, and with a plasmid encoding APP-GFP carrying the double mutation F615P/D664A. After 24-h cells were fixed, permeabilized, stained for EEA1 and TGN46, and examined by fluorescence microscopy. Merging green, red, and blue channels generated the fourth image on each row; yellow indicates overlapping localization of the green and red channels, cyan indicates overlapping localization of the green and blue channels, magenta indicates overlapping localization of the red and blue channels, and white indicates overlapping localization of the red, green, and blue channels. Insets show 2× magnifications. Bar, 10 µm.
(TIF)

Figure S5 **APP redistributes from endosomes to the TGN upon overexpression of μ4-F255A.** H4 neuroglioma cells were cotransfected with a plasmid encoding either of the indicated variants of μ4, and with a plasmid encoding APP-GFP carrying the double mutation F615P/D664A. After 36-h cells were fixed, permeabilized, stained for TGN46 and EEA1, and examined by fluorescence microscopy. Merging green, red, and blue channels generated the fourth image on each row; yellow indicates overlapping localization of the green and red channels, cyan indicates overlapping localization of the green and blue channels, magenta indicates overlapping localization of the red and blue channels, and white indicates overlapping localization of the red, green, and blue channels. Insets show 2× magnifications. Bar, 10 µm.
(TIF)

Table S1 **Distribution of APP-GFP in cells overexpressing μ4 constructs.**
(DOCX)
