# Supplementary figures and images for: Structural and Functional Characterization of Cargo-Binding Sites on the μ4-Subunit of Adaptor Protein Complex 4
Source: PLoS One. 2014 Feb 3;9(2):e88147. doi: 10.1371/journal.pone.0088147 (PMC3912200; doi:10.1371/journal.pone.0088147)

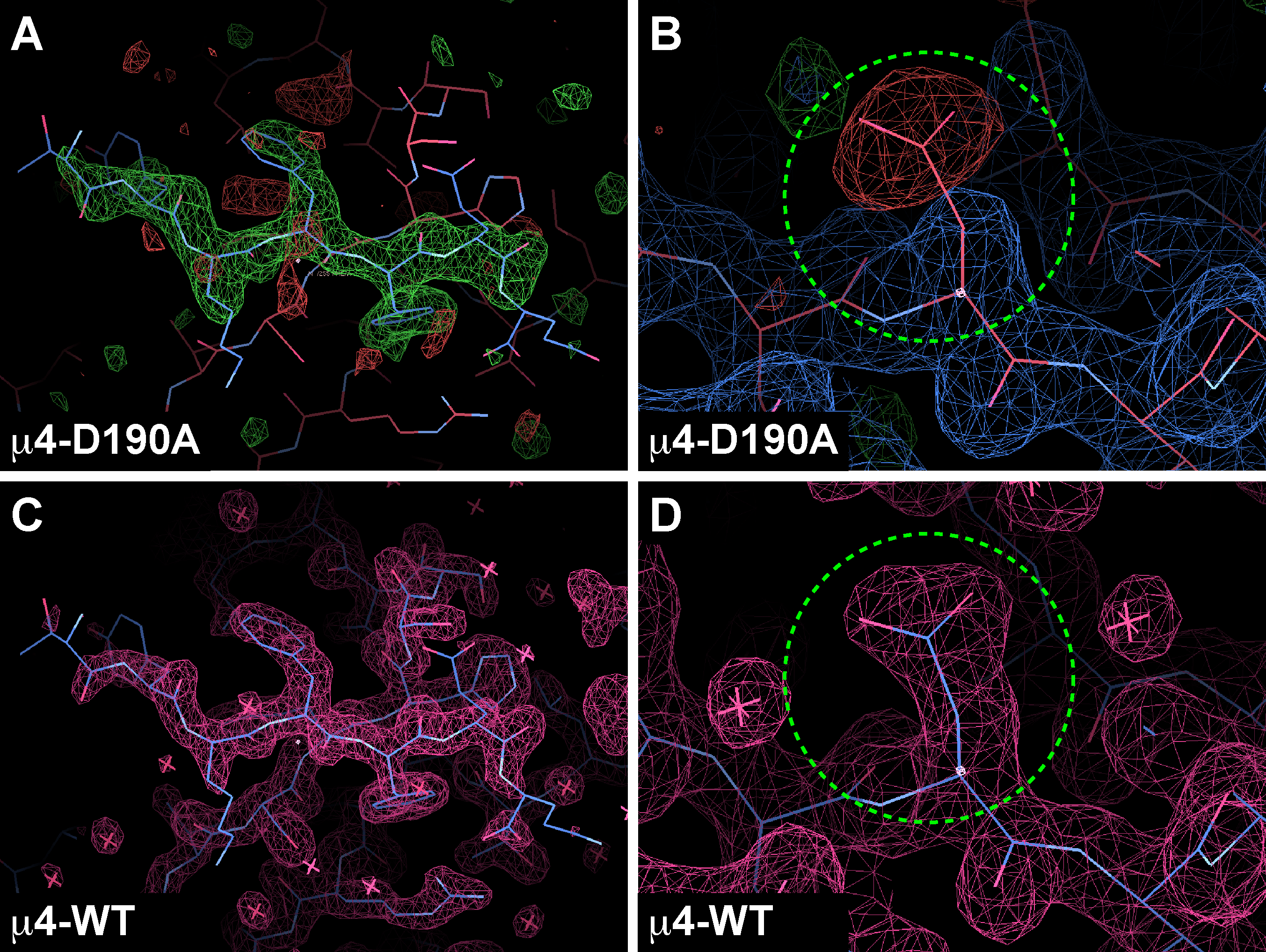

Supplement: Figure S2 — Comparison of electron density maps of μ4 and μ4-D190A. (A) Difference electron density map of the APP peptide (TYKFFEQ) bound to μ4-D190A C-terminal domain (F o-F c contoured at 3σ, green mesh). The density was calculated after solving the structure by molecular replacement using wild-type μ4 C-terminal domain without ligands as search model. The position of the peptide was revealed by superimposing the search model with the μ4 C-terminal domain bound to the APP peptide (represented in stick model; pdb entry 3L81). (B) Negative difference electron density map of μ4-D190A (F o-F c contoured at 3σ, red mesh) at the site of Asp-190 (dotted green circle), observed in the initial electron density as described in (A), superimposed to the electron density map of μ4-D190A (2F o-F c contoured at 2σ, blue mesh) after refining against μ4-D190A. The superimposed structure of 3L81 is shown as sticks. (C) Electron density map and stick model of 3L81 at a similar region shown in (A). (D) Electron density map and stick model of 3L81 at a similar region shown in (B). (TIF) [file pone.0088147.s002.tif]

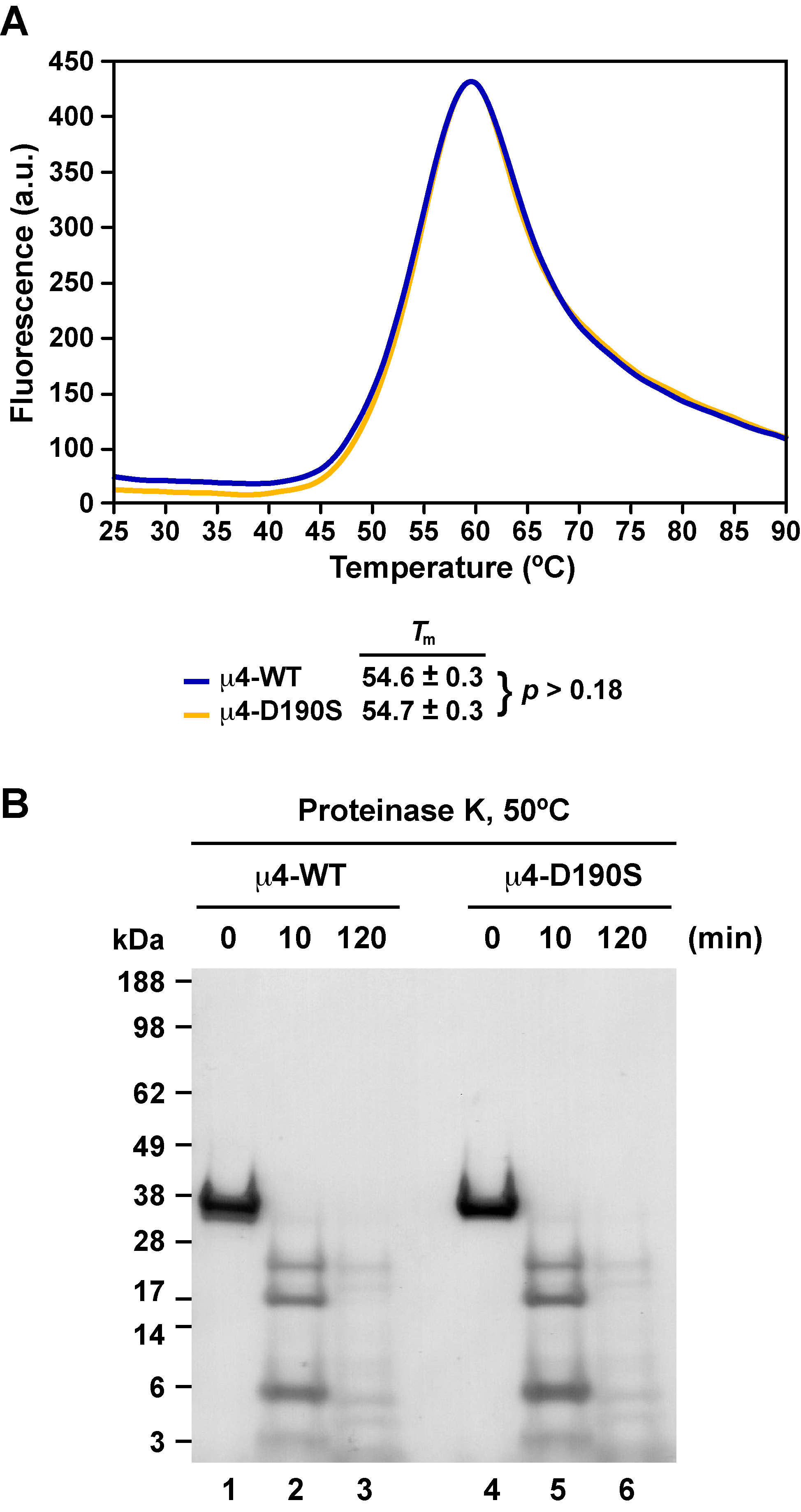

Supplement: Figure S3 — Thermal stability and limited proteolysis analyses of the C-terminal domain of μ4-D190S. (A) The thermal unfolding of the recombinant C-terminal domain of wild-type μ4, or μ4-D190S was analyzed by differential scanning fluorimetry following fluorescence changes in the presence of SYPRO Orange. Representative melting curves of each μ4 variant are shown. The calculated T m value, defined as the maximum of the first derivative of the raw data, is expressed as the mean ± SD (n = 3). (B) Samples of recombinant C-terminal domain of wild-type μ4, or μ4-D190S were incubated with proteinase K at 50°C at an enzyme:substrate ratio of 1∶100, and after the times indicated on top of the panel the digestion was stopped by addition of PMSF. The reaction products were analyzed by SDS-PAGE and gels stained with Coomassie Brilliant Blue. The position of molecular mass markers is indicated on the left. (TIF) [file pone.0088147.s003.tif]

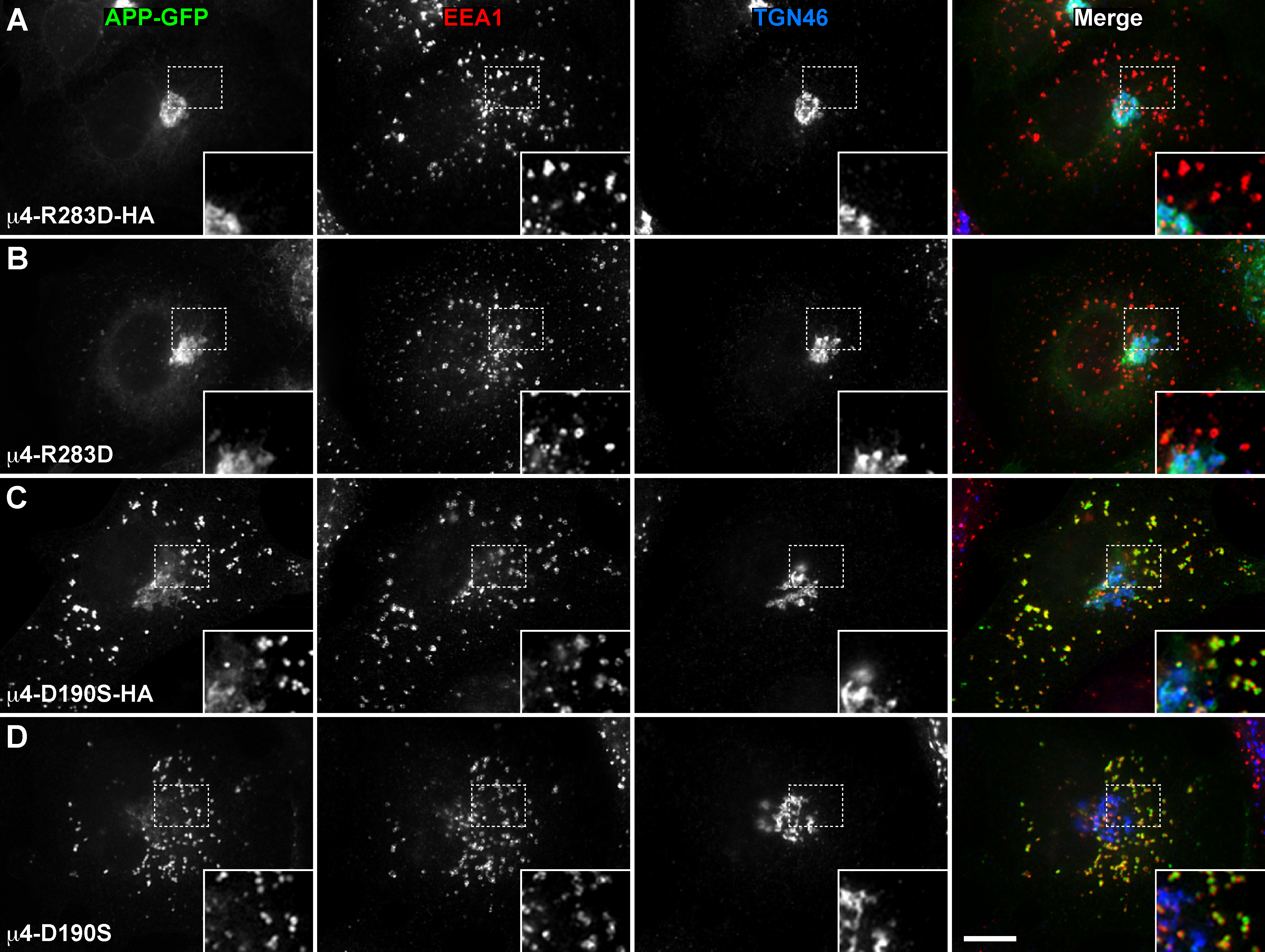

Supplement: Figure S4 — APP redistributes from endosomes to the TGN upon overexpression of μ4-R283D-HA or μ4-R283D. MD-MB-231 cells were cotransfected with a plasmid encoding either of the indicated HA-epitope-tagged or untagged variants of μ4, and with a plasmid encoding APP-GFP carrying the double mutation F615P/D664A. After 24-h cells were fixed, permeabilized, stained for EEA1 and TGN46, and examined by fluorescence microscopy. Merging green, red, and blue channels generated the fourth image on each row; yellow indicates overlapping localization of the green and red channels, cyan indicates overlapping localization of the green and blue channels, magenta indicates overlapping localization of the red and blue channels, and white indicates overlapping localization of the red, green, and blue channels. Insets show 2× magnifications. Bar, 10 µm. (TIF) [file pone.0088147.s004.tif]

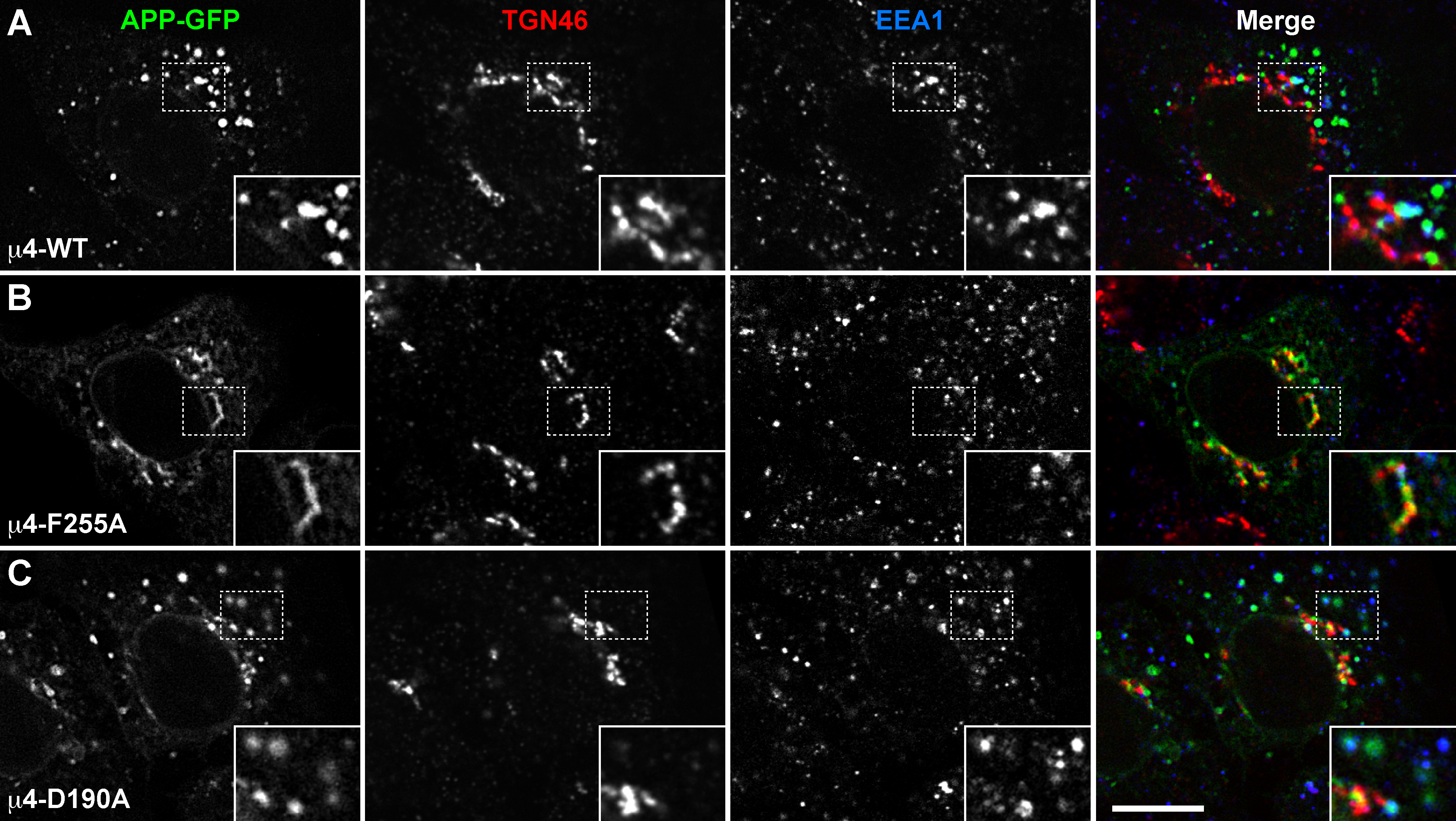

Supplement: Figure S5 — APP redistributes from endosomes to the TGN upon overexpression of μ4-F255A. H4 neuroglioma cells were cotransfected with a plasmid encoding either of the indicated variants of μ4, and with a plasmid encoding APP-GFP carrying the double mutation F615P/D664A. After 36-h cells were fixed, permeabilized, stained for TGN46 and EEA1, and examined by fluorescence microscopy. Merging green, red, and blue channels generated the fourth image on each row; yellow indicates overlapping localization of the green and red channels, cyan indicates overlapping localization of the green and blue channels, magenta indicates overlapping localization of the red and blue channels, and white indicates overlapping localization of the red, green, and blue channels. Insets show 2× magnifications. Bar, 10 µm. (TIF) [file pone.0088147.s005.tif]
