# Supplementary material for: Structural and Functional Characterization of Cargo-Binding Sites on the μ4-Subunit of Adaptor Protein Complex 4
Source: PLoS One. 2014 Feb 3;9(2):e88147. doi: 10.1371/journal.pone.0088147 (PMC3912200; doi:10.1371/journal.pone.0088147)
Supplement: Table S1 — Distribution of APP-GFP in cells overexpressing μ4 constructs. (DOCX) [file pone.0088147.s006.docx]

**Table S1. Distribution of APP-GFP in cells overexpressing μ4 constructs**

| Construct | Localization^a^ | | | n^b^ | *P-value*^c^ | *P-value*^d^ |
| --- | --- | --- | --- | --- | --- | --- |
|  | *Endosomes* | *Golgi/TGN* | *Rest of the cell* |  |  |  |
| μ4-HA | 76.0 ± 3.8 | 12.0 ± 4.2 | 12.0 ± 2.9 | 10 | *p*<0.001 | *p*<0.001 |
| μ4-D190A-HA | 67.1 ± 6.3 | 20.1 ± 6.4 | 12.8 ± 6.0 | 15 | *p*<0.001 |  |
| μ4-D190S-HA | 78.1 ± 8.6 | 10.1 ± 5.6 | 11.8 ± 4.0 | 10 | *p*<0.001 |  |
| μ4-F255A-HA | 11.3 ± 4.1 | 77.9 ± 4.8 | 10.8 ± 2.8 | 15 | *p*<0.001 |  |
| μ4-R283D-HA | 12.9 ± 5.3 | 77.7 ± 7.2 | 9.4 ± 3.1 | 15 | *p*<0.001 |  |
| μ4 wt | 76.5 ± 6.2 | 11.3 ± 8.9 | 12.2 ± 3.0 | 10 | *p*<0.001 | *p*<0.001 |
| μ4-D190A | 64.2 ± 3.5 | 24.1 ± 5.8 | 11.7 ± 5.7 | 15 | *p*<0.001 |  |
| μ4-F255A | 8.1 ± 6.4 | 80.7 ± 5.4 | 11.2 ± 2.3 | 15 | *p*<0.001 |  |

^a^ Expressed as % ± SD of the total APP-GFP signal. For more explanation see ‘Immunofluorescence Microscopy and Quantification of Colocalization’ in ‘Experimental Procedures’.

^b^ Number of cells used for quantification.

^c^ *P-value* obtained comparing *Endosomes* versus *Golgi/TGN* localization.

^d^ *P-value* obtained comparing μ4-HA versus μ4-D190A-HA, and wild-type (wt) μ4 versus μ4-D190A.
